# Silver Nanoparticle-Assisted Laser Desorption/Ionization Mass Spectrometry Imaging of Low-Molecular-Weight Compounds in a Narcissus Bulb

**DOI:** 10.3390/molecules31172941

**Published:** 2026-08-22

**Authors:** Izabela Arendowska, Adrian Arendowski

**Affiliations:** 1 Department of Histology and Embryology, Faculty of Medicine, Medical College, University of Rzeszów, Leszka Czarnego 4, 35-615 Rzeszow, Poland; iarendowska@ur.edu.pl; 2 Faculty of Chemistry, Rzeszów University of Technology, Powstańców Warszawy 6, 35-029 Rzeszow, Poland

**Keywords:** mass spectrometry imaging, narcissus bulb, plant spatial metabolomics, silver nanoparticles, surface-assisted laser desorption/ionization

## Abstract

Surface-assisted laser desorption/ionization mass spectrometry imaging (SALDI-MSI) using steel target coated with silver nanoparticles (AgNPs) by electrodeposition was applied for the direct visualization of metabolites in bulb tissue of *Narcissus pseudonarcissus*. Fresh bulb cross-sections were transferred onto an AgNP-SALDI target by a simple tissue imprint procedure and analyzed using a MALDI TOF mass spectrometer operating in positive-ion reflectron mode. Ion images were generated after total ion current normalization and metabolite annotation was performed based on accurate mass measurements, characteristic silver adduct formation, database searches and literature data. Twenty-one ion images representing seventeen putatively annotated metabolites were selected for detailed discussion. The putatively annotated compounds included primary metabolites (histidine, malic acid, succinic acid, thiamine, coenzyme A and acetyl-coenzyme A), phytohormones (indole-3-acetic acid, indole-3-butyric acid, 3-indolepropionic acid, 4-chloroindole-3-acetic acid and abscisic acid), flavonoids and characteristic *Amaryllidaceae* alkaloids, including galanthamine, lycoramine, crinine, assoanine, habranthine and 5,6-dihydrobicolorine. Distinct spatial distributions were observed for individual metabolites, reflecting the metabolic heterogeneity of bulb tissues. The results demonstrate that AgNPs-SALDI-MSI provides a rapid, matrix-free approach for in situ visualization of low-molecular-weight metabolites in plant tissues while preserving their spatial organization, making it a promising tool for plant metabolomics and phytochemical investigations.

## 1. Introduction

*Narcissus pseudonarcissus* L. (*Amaryllidaceae*), commonly known as wild daffodil or Lent lily, is a perennial geophyte widely distributed in Europe and cultivated as an ornamental and medicinal plant [1]. Its underground bulb is a specialized storage organ responsible for the accumulation of reserve metabolites, regulation of dormancy and rapid re-initiation of growth in favourable environmental conditions [2]. Bulbs of *Narcissus* species are also characterised by the presence of structurally diverse *Amaryllidaceae* alkaloids, which constitute a distinctive chemotaxonomic feature of the family and play an important role in plant defence against herbivores and pathogens [3]. Among them, galanthamine has gained particular attention due to its clinical application in the treatment of Alzheimer’s disease, which has stimulated extensive phytochemical investigations of *Narcissus* tissues [4].

In addition to specialized metabolites, the bulb contains phytohormones and primary metabolites that regulate the transition between dormancy and active growth [5,6]. Auxins and abscisic acid are key regulators of meristem activation, root initiation and sprouting processes, while organic acids and cofactors reflect the metabolic readiness of the tissue despite its quiescent physiological state [7,8]. Therefore, the bulb represents a highly organised metabolic system in which defence, energy metabolism and developmental regulation coexist within a limited anatomical space.

The chemical composition of *Narcissus* has been studied using chromatographic and spectroscopic techniques such as GC–MS [9,10], LC–MS [11], HPLC [12] and NMR [13]. Although these methods provide detailed qualitative and quantitative information, they require extraction of analytes from the tissue, resulting in the loss of spatial information about metabolite localization. In contrast, mass spectrometry imaging (MSI) enables the direct visualisation of molecular distributions in biological samples without labelling and with simultaneous detection of multiple compound classes [14,15]. MSI has already been successfully applied to plant tissues to study the spatial organisation of primary and specialized metabolism as well as the localisation of defence compounds and phytohormones [16,17].

Matrix-assisted laser desorption/ionization (MALDI) is currently the most widely used ionization technique in MSI experiments. It allows imaging of proteins, lipids and metabolites in plant tissues [18]; however, the use of organic matrices is associated with several limitations, particularly in the low *m*/*z* range, including matrix-related background signals, inhomogeneous crystallisation and restricted spatial resolution [19]. These drawbacks are especially critical for the analysis of low-molecular-weight plant metabolites such as phytohormones, organic acids and alkaloids.

Surface-assisted laser desorption/ionization (SALDI) employing nanostructured targets has emerged as an alternative approach that can overcome many of these limitations by enabling matrix-free ionization with reduced background and improved reproducibility [20,21]. In particular, metal nanoparticle-enhanced targets provide efficient energy transfer and promote the formation of characteristic metal adduct ions, which is advantageous for the detection of small molecules. Silver nanoparticle-enhanced surfaces can promote the ionization of a broad range of low-molecular-weight compounds, while silver adduct formation may provide additional information useful for metabolite annotation [21,22].

Nanoparticle-assisted SALDI has already been successfully applied to MSI of plant tissues and small biological objects, including the visualisation of endogenous metabolites in onion bulb cross-sections and the spatial analysis of plant specialized metabolites [23,24,25]. However, spatial metabolomics of the *Narcissus* bulbs remains largely unexplored, particularly in the context of direct imprint analysis, which allows rapid sample preparation while preserving the chemical organisation of the tissue.

The aim of the present study was to apply AgNPs-assisted SALDI-MSI for the spatially resolved analysis of low-molecular-weight metabolites in the bulb of *Narcissus pseudonarcissus*. Special attention was paid to the localisation of *Amaryllidaceae* alkaloids, phytohormones and central metabolic intermediates, and to the correlation of their spatial distribution with the physiological function of the bulb as a storage and survival organ.

## 2. Results and Discussion

In order to present capabilities of the SALDI target with silver nanoparticles electrodeposited on stainless steel plate for direct analysis of intact plant material, the bulb of *Narcissus pseudonarcissus* was selected as a model biological object. The narcissus bulb is a perennial underground storage organ composed of a compressed stem (basal plate) surrounded by concentric fleshy scale leaves that accumulate reserve compounds required for dormancy maintenance and subsequent plant regeneration. Besides carbohydrates, the bulb is known to contain numerous low-molecular-weight metabolites, including *Amaryllidaceae* alkaloids, phytohormones and intermediates of primary metabolism, making it an attractive model for spatial metabolomic investigations.

**Figure 1:**
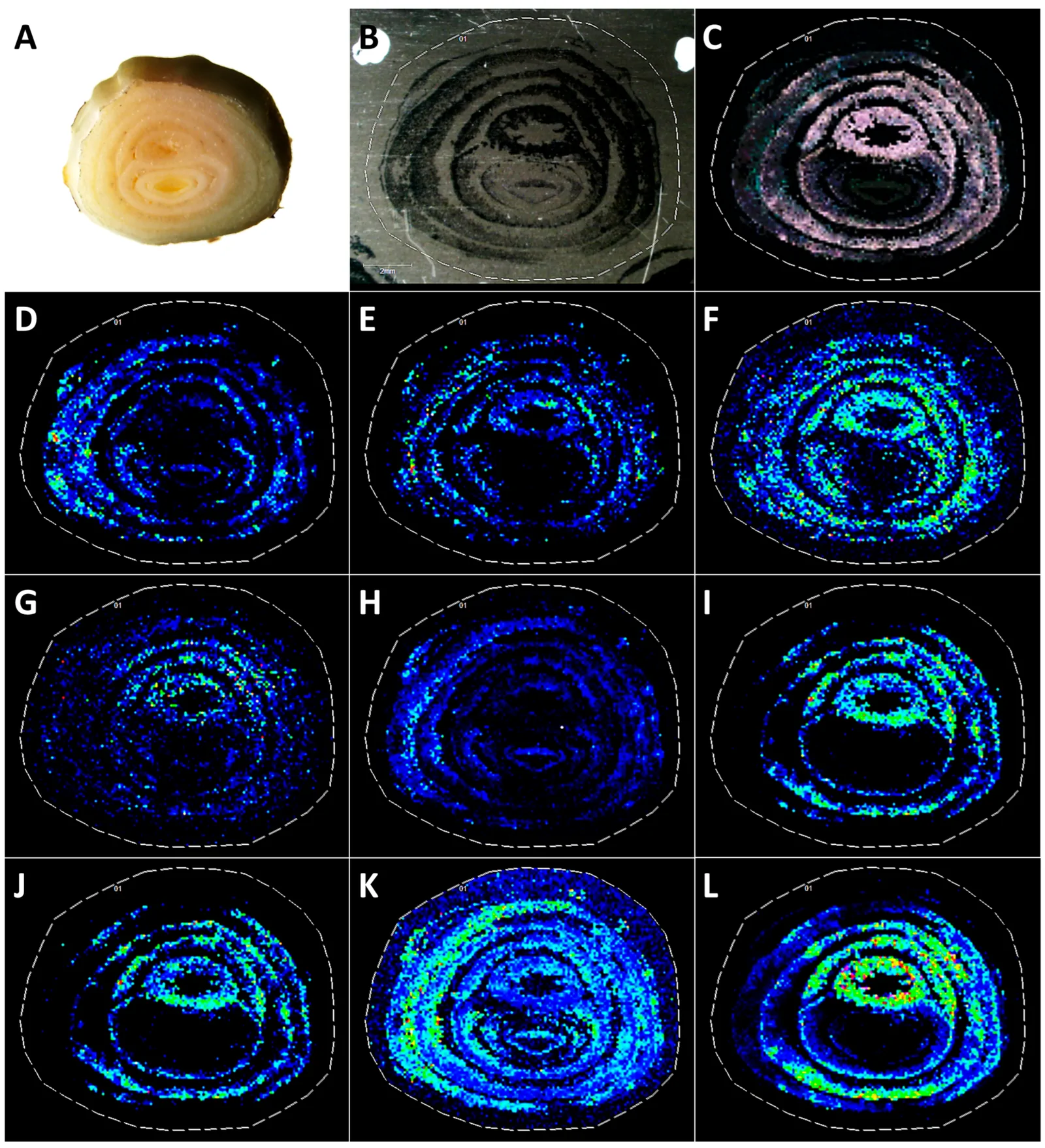

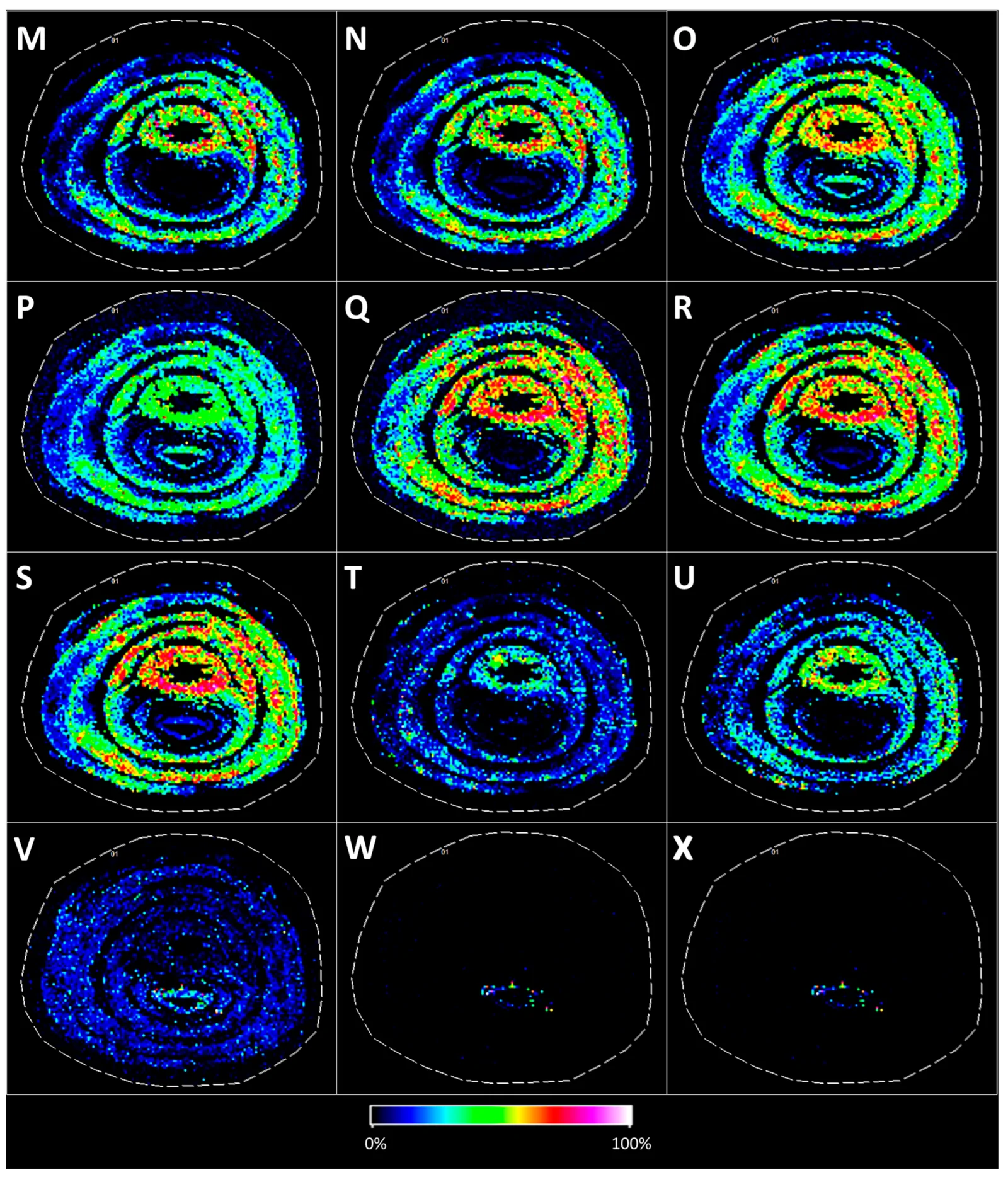
Results of MS imaging. Optical photograph of the imaged surface of the narcissus bulb (**A**) and its imprint on the AgNPs-SALDI target (**B**). Image (**C**) (TIC normalized) contain ion image for all twenty-one *m*/*z* values. Images (**D**–**L**) (TIC normalized) contains ion images for *m*/*z* values of 156.0768 (**D**), 172.9852 (**E**), 176.0706 (**F**), 190.0863 (**G**), 204.1019 (**H**), 224.9311 (**I**), 226.9308 (**J**), 240.1019 (**K**) and 247.9875 (**L**), respectively. Spatial resolution of ion images was 100 μm × 100 μm. Mass tolerance used for image generation was ±0.001%. Results of MS imaging. Images (**M**–**X**) (TIC normalized) contains ion images for *m*/*z* values of 265.1440 (**M**), 268.1332 (**N**), 272.1281 (**O**), 273.0763 (**P**), 281.9679 (**Q**), 283.9675 (**R**), 288.1594 (**S**), 312.1570 (**T**), 372.0168 (**U**), 768.1225 (**V**) 916.0302 (**W**) and 918.0299 (**X**), respectively. Spatial resolution of ion images was 100 μm × 100 μm. Mass tolerance used for image generation was ±0.001%.

A cross-section of the Narcissus bulb (Figure 1A) was gently pressed onto the AgNPs-SALDI target and subsequently removed, leaving a tissue imprint on the nanoparticle-coated surface (Figure 1B). Mass spectrometry imaging was then performed directly on the imprint using a spatial resolution of 100 μm × 100 μm. The applied spatial resolution enabled visualization of low-molecular-weight metabolite distributions across the imprint at the scale of major tissue regions, while maintaining satisfactory signal intensity and acquisition time. The optical image of the bulb cross-section and the corresponding imprint deposited on the AgNPs-SALDI target are shown in Figure 1A and Figure 1B, respectively. The morphological correspondence between the tissue section and the resulting imprint indicates that the imprinting procedure preserved the overall spatial organization of the sampled tissue. However, this optical correspondence does not provide a quantitative measure of metabolite transfer efficiency. Imaging of the tissue imprint yielded a total of 11,645 mass spectra. These spectra represent spatially resolved sampling points from a single biological specimen and should therefore not be interpreted as biological replicates. Accordingly, the present dataset does not permit assessment of biological variability or statistical reproducibility across independent bulbs. Prior to data interpretation, a list of more than 500 candidate metabolites expected to occur in the narcissus bulb tissue was compiled from metabolomic databases and the literature. This list included amino acids, organic acids, phytohormones, *Amaryllidaceae* alkaloids and other primary and specialized metabolites. Among the detected ions, twenty-one metabolite signals (Figure 1, Table 1) were selected for detailed discussion based on their analytical quality, biological relevance and chemotaxonomic significance. These ion images represent the spatial distribution of seventeen putatively identified metabolites across the bulb imprint. Features that could not be confidently assigned to specific metabolites based on the available accurate-mass and database/literature evidence were not assigned compound names and were excluded from the detailed interpretation of individual metabolites. Such unannotated molecular features may nevertheless represent potentially relevant compounds and should not be considered biologically irrelevant solely because they are absent from the currently available reference databases.

The transverse section displayed a pronounced concentric organization corresponding to the fleshy scale tissues of the bulb. Several molecular features showed distributions that followed this general tissue organization, whereas others exhibited more heterogeneous spatial patterns. Because the present analysis was performed on a transverse section without histological reference staining, specific anatomical structures such as the basal plate and meristematic region could not be unambiguously delineated. Therefore, the observed molecular distributions are interpreted at the level of major tissue regions rather than specific anatomical structures.

The summed ion image obtained after total ion current (TIC) normalization (Figure 1C) represents the distribution of all selected ions listed in Table 1. The image reveals a pronounced chemical heterogeneity within the imprint area, indicating that the metabolic composition of the bulb tissue is spatially differentiated. Such heterogeneity is expected for storage organs in geophytic plants, where metabolic activity may differ between distinct tissue regions and functional domains [26]. A total of twenty-one ions were selected for further analysis based on their signal intensity and reproducibility across the analyzed area.

**Table 1:** MSI analysis results of the *Narcissus pseudonarcissus* bulb imprint on silver nanoparticles SALDI target. Mass extraction window ±0.001% (±10 ppm).

Observed *m*/*z*	Proposed Ion	Putative Annotation	Spatial Image	Reference
156.0768	[C_6_H_9_N_3_O_2_+H]^+^	Histidine	Figure 1D	[27]
172.9852	[C_4_H_6_O_5_+K]^+^	Malic acid	Figure 1E	[28]
176.0706	[C_10_H_9_NO_2_+H]^+^	Indole-3-acetic acid (IAA)	Figure 1F	[29]
190.0863	[C_11_H_11_NO_2_+H]^+^	3-Indolepropionic acid (IPA)	Figure 1G	[30]
204.1019	[C_12_H_13_NO_2_+H]^+^	Indole-3-butyric acid (IBA)	Figure 1H	[31]
224.9311	[C_4_H_6_O_4_+^107^Ag]^+^	Succinic acid	Figure 1I	[28]
226.9308	[C_4_H_6_O_4_+^109^Ag]^+^		Figure 1J	
240.1019	[C_15_H_13_NO_2_+H]^+^	5,6-Dihydrobicolorine	Figure 1K	[32]
247.9875	[C_10_H_8_ClNO_2_+K]^+^	4-Chloroindole-3-acetic acid (4-Cl-IAA)	Figure 1L	[33]
265.1440	[C_15_H_20_O_4_+H]^+^	Abscisic acid (ABA)	Figure 1M	[7]
268.1332	[C_17_H_17_NO_2_+H]^+^	Assoanine	Figure 1N	[1]
272.1281	[C_16_H_17_NO_3_+H]^+^	Crinine	Figure 1O	[1]
273.1359	[C_16_H_18_NO_3_+H]^+^	Habranthine	Figure 1P	[1]
281.9679	[C_10_H_9_NO_2_+^107^Ag]^+^	Indole-3-acetic acid (IAA)	Figure 1Q	[29]
283.9675	[C_10_H_9_NO_2_+^109^Ag]^+^		Figure 1R	
288.1594	[C_17_H_21_NO_3_+H]^+^	Galanthamine	Figure 1S	[1]
312.1570	[C_17_H_23_NO_3_+Na]^+^	Lycoramine	Figure 1T	[1]
372.0168	[C_12_H_17_N_4_OS+^107^Ag]^+^	Thiamine	Figure 1U	[34]
768.1225	[C_21_H_36_N_7_O_16_P_3_S+H]^+^	Coenzyme A	Figure 1V	[35]
916.0302	[C_23_H_38_N_7_O_17_P_3_S+^107^Ag]^+^	Acetyl coenzyme A	Figure 1W	[36]
918.0299	[C_23_H_38_N_7_O_17_P_3_S+^109^Ag]^+^		Figure 1X	

Several annotated ions correspond to primary metabolites involved in central plant metabolism (Table 1). Among them, histidine (*m*/*z* 156.0768, Figure 1D) represents a nitrogen-containing amino acid associated with protein biosynthesis and nitrogen storage in plant tissues [27]. Its relatively uniform spatial distribution across the imprint suggests that amino acid pools are broadly present in the storage parenchyma of the bulb.

Organic acids associated with the tricarboxylic acid (TCA) cycle were also putatively identified, including malic acid (*m*/*z* 172.9852, Figure 1E) and succinic acid observed as characteristic silver adduct ions (*m*/*z* 224.9311 and 226.9308 corresponding to ^107^Ag and ^109^Ag adducts, Figure 1I,J). These compounds play a central role in cellular respiration and carbon metabolism [28]. Their relatively diffuse spatial distribution in the MSI images indicates that respiratory metabolism remains active across large regions of the bulb tissue. This observation supports the view that bulbs, although physiologically dormant, maintain basal metabolic activity necessary to preserve cellular viability and to enable rapid resumption of growth under favourable environmental conditions [26].

Additional metabolites putatively annotated in this experiment include thiamine (vitamin B1; *m*/*z* 372.0168, Figure 1U), coenzyme A (*m*/*z* 768.1225, Figure 1V) and acetyl-coenzyme A (*m*/*z* 916.0302 and 918.0299, Figure 1W,X). These molecules are key cofactors involved in enzymatic reactions controlling energy metabolism, fatty acid synthesis and carbon flux regulation in plant cells [35]. Their presence in the bulb tissue further supports the notion that the storage organ retains a metabolically competent state even during the dormant phase.

Unlike several other primary metabolites exhibiting relatively diffuse distributions, acetyl-coenzyme A (*m*/*z* 916.0302 and 918.0299; Figure 1W,X) displayed a distinctly localized spatial pattern within the bulb imprint. Such heterogeneous distribution is consistent with the central metabolic role of acetyl-CoA as a key branching intermediate linking glycolysis, β-oxidation of fatty acids and the tricarboxylic acid cycle with numerous anabolic pathways, including fatty acid, isoprenoid and phenylpropanoid biosynthesis [36]. Rather than being uniformly distributed throughout the storage tissue, acetyl-CoA is expected to accumulate preferentially in metabolically active cells exhibiting high mitochondrial and plastidial activity.

In geophytic storage organs, localized pools of acetyl-CoA may therefore reflect regions maintaining elevated metabolic competence during dormancy, potentially reflecting metabolically active regions involved in growth and organ regeneration following dormancy release [36,37]. Acetyl-CoA also provides carbon skeletons required for membrane lipid biosynthesis accompanying rapid cell proliferation during sprouting. Consequently, its restricted localization observed in the ion images may indicate metabolic pre-patterning of developmental centres within the bulb rather than simple storage of this metabolite.

In addition to its metabolic function, acetyl-CoA has recently emerged as an important signalling metabolite controlling epigenetic regulation through histone acetylation. Local fluctuations in acetyl-CoA availability have been shown to influence gene expression associated with developmental transitions and environmental responses in plants [38,39]. Although the present MSI experiment does not allow direct assessment of subcellular compartmentation, the observed spatial localization suggests that acetyl-CoA metabolism is tightly regulated within specific functional domains of the narcissus bulb.

Interestingly, the localization of acetyl-CoA was considerably more restricted than that of coenzyme A (Figure 1V), suggesting that the intracellular conversion of CoA into acetyl-CoA occurs preferentially in metabolically active tissue regions rather than throughout the entire storage parenchyma. This observation is consistent with the dynamic regulation of acetyl-CoA production by pyruvate dehydrogenase and β-oxidation pathways during the transition from dormancy to active growth [40].

Several detected ions correspond to plant growth regulators or related indole derivatives. Indole-3-acetic acid (IAA), the principal auxin in higher plants, was observed at *m*/*z* 176.0706 (Figure 1F) as a protonated ion and additionally as characteristic silver adduct ions at *m*/*z* 281.9679 and 283.9675 (Figure 1Q,R). The identical spatial distributions observed for the protonated and silver-adduct forms support the reliability of this assignment. IAA plays a fundamental role in regulating cell elongation, meristem activity and organ initiation [29]. The MSI images revealed localized regions of enhanced IAA signal intensity, suggesting spatially heterogeneous distribution of the putatively identified auxin signal within the bulb tissue. Such spatial heterogeneity in auxin distribution may be associated with the regulation of developmental processes, including root formation and shoot emergence during bulb sprouting.

Related indole compounds were also putatively annotated, including indole-3-butyric acid (IBA; *m*/*z* 204.1019, Figure 1H) and 3-indolepropionic acid (IPA; *m*/*z* 190.0863, Figure 1G). IBA is commonly regarded as an auxin precursor or storage form that can be converted into IAA through β-oxidation pathways [31]. IPA may represent a metabolite associated with indole metabolism or auxin catabolism [30]. The spatial patterns observed for these metabolites partially overlap with the IAA signal but also show distinct localization domains, suggesting that multiple indole-related metabolic pathways are active within the bulb.

Another hormone was putatively annotated in the imaging experiment as abscisic acid (ABA; *m*/*z* 265.1440, Figure 1M). ABA is widely recognized as a key regulator of dormancy and stress responses in plants [7]. The ion signal tentatively assigned to ABA within the bulb tissue is consistent with its physiological role in maintaining the dormant state and controlling the transition to active growth when environmental conditions become favourable.

A chlorinated auxin analogue, 4-chloroindole-3-acetic acid (4-Cl-IAA; *m*/*z* 247.9875, Figure 1L), was also tentatively identified. Although this compound has been reported primarily in specific plant species, chlorinated auxins are known to exhibit strong biological activity and may participate in developmental regulation [33].

The most characteristic metabolites putatively annotated in the bulb tissue belong to the group of *Amaryllidaceae* alkaloids, which represent a distinctive class of specialized metabolites largely restricted to members of the *Amaryllidaceae* family and are widely recognized as valuable chemotaxonomic markers [1]. In the present experiment, several alkaloids previously reported in *Narcissus* species or closely related members of the family were tentatively annotated, including 5,6-dihydrobicolorine (*m*/*z* 240.1019, Figure 1K) [32], assoanine (*m*/*z* 268.1332, Figure 1N), crinine (*m*/*z* 272.1281, Figure 1O), habranthine (*m*/*z* 273.1359, Figure 1P), galanthamine (*m*/*z* 288.1594, Figure 1S) and lycoramine, observed as the sodium adduct (*m*/*z* 312.1570, Figure 1T). Together, these compounds represent several structural subclasses of *Amaryllidaceae* alkaloids, reflecting the characteristic metabolic diversity of the narcissus bulbs [1].

The ion images corresponding to these metabolites revealed heterogeneous spatial distributions, with distinct regions of enhanced signal intensity rather than uniform signal distribution across the bulb imprint. Although the spatial patterns differed between individual alkaloids, all compounds displayed distinct spatially localized signals, suggesting that their distribution within the bulb may be associated with tissue-specific differences in biosynthesis, transport and/or storage. Such compartmentalization has previously been proposed for *Amaryllidaceae* alkaloids, whose biosynthesis is believed to occur in metabolically active tissues followed by translocation and accumulation in specialized storage cells or vacuoles [41,42]. This spatial organization is consistent with the physiological role of bulbs as perennial storage organs that require efficient chemical protection against soil-borne pathogens and herbivores.

Among the alkaloids putatively annotated in the present study, galanthamine is of particular interest because of its well-established pharmaceutical application as a reversible acetylcholinesterase inhibitor used in the symptomatic treatment of Alzheimer’s disease [43]. The detection of this compound directly in the bulb imprint demonstrates the capability of the AgNPs-SALDI-MSI approach to visualize pharmaceutically relevant natural products in situ within plant tissues without prior extraction or chromatographic separation. Moreover, the simultaneous detection of lycoramine, another galanthamine-type alkaloid reported from several Narcissus species, further supports the ability of the proposed analytical approach to reveal the spatial organization of structurally related *Amaryllidaceae* alkaloids. Such direct localization of bioactive metabolites represents a valuable advantage over conventional extract-based analytical techniques, which provide comprehensive chemical profiles but do not preserve information on tissue-specific distribution.

The simultaneous visualization of multiple representatives of different *Amaryllidaceae* alkaloid skeletons further highlights the suitability of AgNPs-SALDI-MSI for investigating the spatial organization of specialized metabolism in geophytic plants. Although the metabolite annotations presented here should be regarded as tentative, the observed ion distributions provide valuable insight into the localization of pharmacologically important metabolites and demonstrate the potential of silver nanoparticle-assisted SALDI imaging for studying alkaloid biosynthesis, accumulation and compartmentalization directly in plant tissues.

Metabolite identification was based on accurate mass measurements, isotopic patterns, database searches and literature data (Table 1). The observed mass errors were within the ±0.001% window used for image extraction. Characteristic isotopic doublets corresponding to ^107^Ag and ^109^Ag adducts further supported the assignment of several compounds, particularly organic acids and auxin derivatives.

Metabolite annotation was based on accurate mass measurements, characteristic isotopic patterns, database searches (METLIN, ChEBI, KEGG, KNApSAcK and MetaCyc) and comparison with literature reports describing the phytochemical composition of *Narcissus* species (Table 1). The confidence of the proposed metabolite assignments varies depending on the available analytical evidence and should therefore be interpreted with appropriate caution. The presence of literature reports for these compounds in *Narcissus* or closely related *Amaryllidaceae* species provides biological support for the proposed annotations but does not constitute definitive structural identification [1]. For several metabolites, including succinic acid and indole-3-acetic acid, the observation of characteristic ^107^Ag/^109^Ag adduct pairs provided additional evidence supporting the proposed annotations. However, in the absence of measurements of authentic reference standards under identical experimental conditions, these isotope patterns should be regarded as supporting rather than definitive evidence for the proposed metabolite assignments. In contrast, the identification of ubiquitous primary metabolites, such as amino acids, organic acids, vitamins and metabolic cofactors, is based primarily on accurate mass measurements and database matching. Owing to the absence of tandem mass spectrometry experiments and analyses of authentic reference standards performed under identical analytical conditions, the reported metabolite identities should be considered tentative annotations rather than unambiguous structural identifications. Accurate mass measurements alone are insufficient to distinguish many isobaric or isomeric metabolites. Because the present workflow was based on direct MSI without chromatographic separation and MS/MS acquisition, the proposed assignments cannot exclude structurally related isomers. Consequently, unambiguous structural identification would require confirmation using tandem MS, chromatographic separation and/or authentic reference standards. Features that cannot be matched to entries in the currently available databases should likewise be regarded as unannotated molecular features rather than excluded a priori as irrelevant. Their structural characterization would require additional analytical evidence, particularly tandem mass spectrometry, comparison with spectral libraries and, where possible, authentic reference standards. Accordingly, although the confidence of annotation for individual compounds may broadly correspond to different confidence levels commonly used in metabolomics, no formal assignment of identification levels was made in the present study [44].

The application of silver nanoparticle-assisted SALDI provided several analytical advantages compared with conventional MALDI imaging. First, the absence of an organic matrix significantly reduced background signals in the low mass region, which is critical for the analysis of small plant metabolites. Second, the formation of characteristic [M+Ag]^+^ adduct ions facilitated the detection and annotation of molecules containing heteroatoms or π-electron systems [22].

The presence of two naturally occurring silver isotopes (^107^Ag and ^109^Ag) generated distinctive isotopic patterns that increased the confidence of compound assignments. Additionally, the nanoparticle-coated surface ensured efficient laser energy absorption and reproducible ion generation across the analyzed area [21].

The use of a simple tissue imprint rather than cryosectioned samples also simplified sample preparation and avoided the need for direct analysis of a physically sectioned tissue surface, making the approach particularly suitable for rapid metabolomic screening of small plant organs [19]. Moreover, tissue imprinting may reduce some sources of analyte redistribution associated with conventional tissue processing. However, the transfer of metabolites from the tissue to the target surface is inherently dependent on the physicochemical properties of individual compounds and the composition of the sampled tissue. Consequently, compound-dependent differences in transfer efficiency, incomplete transfer, and potential lateral diffusion may affect spatial fidelity and should be considered when interpreting MSI data obtained using this approach. As a direct comparison between tissue imprinting and cryosection-based MSI was not included in the present study, the relative advantages and limitations of these approaches cannot be quantitatively established from the current dataset. Such a comparison would therefore represent an important direction for future methodological validation. Although a 3 s imprinting time was successfully applied in previous nanoparticle-assisted MSI studies of plant tissues [23,24,25], including ^109^AgNPs-LDI-MSI of *Aloe vera* leaves [45], the present study did not include a systematic evaluation of different contact times. Therefore, 3 s should not be considered a universally optimized condition. Differences in tissue composition and physicochemical properties of individual metabolites may influence transfer efficiency and spatial fidelity, and systematic optimization of imprinting time remains necessary for broader methodological validation.

The ion intensities observed in the MSI data should be interpreted as relative molecular signal distributions rather than direct measures of metabolite concentrations. Ion signal intensity may be influenced by several factors, including compound-dependent ionization efficiency, local surface properties, tissue composition and differences in analyte transfer during imprinting. Therefore, differences in ion intensity between tissue regions cannot be directly interpreted as quantitative differences in metabolite concentration. Absolute or validated quantitative measurements were beyond the scope of the present proof-of-concept study.

The MSI results obtained in this study provide insights into the metabolic organization of the Narcissus bulb. The coexistence of primary metabolites related to respiration, phytohormones controlling growth and dormancy, and defensive alkaloids illustrates the multifunctional nature of the bulb as a storage and survival organ [5].

The relatively homogeneous distribution of central metabolic intermediates suggests that the storage parenchyma maintains a basal level of metabolic activity even during dormancy. In contrast, the more localized distribution of alkaloids and phytohormones indicates that these compounds are subject to spatial regulation within the tissue [42]. Such metabolic compartmentalization may play an important role in coordinating defence mechanisms with developmental processes leading to sprouting and organ formation.

The spatial distribution of several molecular features appeared to follow the general organization of the bulb tissue, suggesting that the imprint-based approach retained information on metabolite localization at the regional tissue scale. However, the present dataset does not provide sufficient anatomical resolution to establish specific associations with structures such as the basal plate or meristematic tissue. Such assignments would require complementary histological characterization or higher-resolution MSI. Therefore, the spatial patterns observed in the present study should be interpreted in relation to the major morphological regions visible in the tissue section rather than as evidence of metabolite localization within specific anatomical structures.

The spatial resolution of 100 µm × 100 µm was sufficient to visualize metabolite distributions across major anatomical regions of the bulb; however, it is insufficient to resolve individual cells, single-cell layers or fine vascular structures. Consequently, the spatial patterns reported here should be interpreted at the tissue and regional anatomical scale rather than at the cellular level. Higher spatial resolution would be required to investigate metabolite distributions in relation to individual cell types or fine anatomical structures.

The present study was based exclusively on transverse bulb sections; therefore, the obtained images provide a two-dimensional representation of metabolite distributions within a single anatomical plane. Longitudinal imaging would provide complementary information on metabolite gradients along the apical–basal axis of the bulb and should be considered in future studies.

## 3. Materials and Methods

### 3.1. Materials

Silver trifluoroacetate (AgTFA) used in nanoparticle synthesis was of 98% purity (Sigma Aldrich, St. Louis, MO, USA). Acetonitrile and 2-propanol were of LC-MS quality and purchased from Sigma-Aldrich (Steinheim, Germany). Optical photographs were made with the use of an Olympus SZX16 microscope (Hamburg, Germany) equipped with Olympus EP50 digital camera (Hamburg, Germany).

### 3.2. Preparation of the AgNPs-SALDI Target

The AgNPs-SALDI target was prepared according to the previously reported electrodeposition procedure [46]. Detailed morphological and physicochemical characterization of the resulting silver nanostructured surface, including electron microscopy analysis, has been reported previously [46]. The AgNPs-SALDI target was prepared from plate of AISI 430 grade stainless steel (Cyfronika s.c., Kraków, Poland) with dimensions 75 × 25 × 0.7 mm (locally made). The two steel plates were placed facing each other in a 100 mL beaker and connected to a Consort EV202 (Turnhout, Belgium) power supply. 20 mg of AgTFA was dissolved in 90 mL of a mixture of 80% isopropanol and 20% acetonitrile and was poured into a beaker. Electrodeposition was carried out at a voltage of 10 V for 15 min. The negative electrode was then cleaned with cotton wool and washed three times in boiling isopropanol and acetonitrile. The AgNPs-SALDI target was used with Bruker MTP Slide—Adapter II (Bruker Daltonics GmbH, Bremen, Germany).

### 3.3. Tissue Preparation

The narcissus bulb was obtained from an amateur home garden in Rzeszów, Podkarpackie Voivodeship, Poland. The bulbs used to grow the plant were purchased from the online shop https://cebulki-kwiatowe.pl/ accessed on 24 July 2026. A small *Narcissus pseudonarcissus* bulb (ca. 10 mm diameter) was washed with deionized water. The tissue was then cut perpendicularly to apex-root axis with a razor blade. The bulb cross-section was then immediately pressed gently against cellulose filter paper three times to remove excess liquid and subsequently brought into contact with the AgNPs-SALDI target using light pressure for 3 s. The 3 s contact time was selected based on preliminary experimental observations and previous reports of short-duration tissue imprinting for nanoparticle-assisted plant MSI [23,24,25,45].

### 3.4. MSI Experiment

Measurements were performed with a Bruker AutoFlex Speed time-of-flight mass spectrometer (Bruker Daltonics GmbH, Bremen, Germany) in positive-ion reflectron mode. The instrument was equipped with a solid-state laser—SmartBeam II 1 kHz, 355 nm. Laser pulse energy was approximately 170 μJ and laser pulse repetition rate was 1000 Hz. The imaging experiment was performed at 100 μm × 100 μm resolution with 500 laser shots per individual spot and with a default random walk applied.

Spectra were recorded in the *m*/*z* range of 100–1500, with ions below *m*/*z* 95 deflected from the flight trajectory. In reflectron mode, the operating voltages were as follows: ion source 1, 19 kV; ion source 2, 16.7 kV; lens 8.4 kV; reflector 1, 21 kV; reflector 2, 9.55 kV. The delay time was 150 ns. Spectra were calibrated on 14 points using internal standards (silver ions and clusters from Ag^+^ to Ag_9_^+^).

### 3.5. Analysis of the Results

FlexImaging 4.0 program (Bruker Daltonics GmbH) was used to analyze the imaging results. All ion images were generated using a ±0.001% *m*/*z* (10 ppm) extraction window and total ion current (TIC) normalization was used throughout. MS data analysis was aided by metabolic databases: METLIN [47], ChEBI [48], KEGG [49], KNApSAcK Core System [50] and MetaCyc [51]. A schematic overview of the experimental workflow is provided in Supplementary Materials Figure S1.

## 4. Conclusions

Silver nanoparticle-assisted SALDI-MSI enabled direct imaging of endogenous metabolites in *Narcissus pseudonarcissus* bulb tissue using a simple imprint preparation procedure without extraction or chromatographic separation. The approach provided high-quality ion images for low-molecular-weight metabolites while avoiding the spectral interferences commonly associated with conventional organic MALDI matrices. The proposed methodology allowed simultaneous visualization of metabolites representing different biochemical pathways, including amino acids, organic acids, phytohormones, vitamins, metabolic cofactors and *Amaryllidaceae* alkaloids. Several compounds exhibited distinct localization patterns, indicating spatial heterogeneity of metabolism within the bulb and illustrating the capability of MSI to preserve biochemical information that is inaccessible using extract-based analytical techniques. Particularly noteworthy was the direct visualization of pharmacologically important alkaloids, including galanthamine and lycoramine, together with plant growth regulators and central metabolic intermediates. These findings demonstrate the feasibility of applying AgNPs-SALDI-MSI for spatial visualization of selected low-molecular-weight compounds in *Narcissus pseudonarcissus* bulb tissue and highlight its potential as a rapid analytical approach for spatial metabolomics of plant tissues.

Several limitations should, however, be considered when interpreting the present results. First, the majority of metabolite assignments were putative and were not comprehensively confirmed by tandem mass spectrometry or authentic reference standards. Second, the study was performed using a single bulb and therefore does not provide information on biological variability or statistical reproducibility. Third, no absolute or fully validated quantitative measurements of metabolite concentrations were performed. Finally, although tissue imprinting offers a simple sample-preparation workflow, the transfer of metabolites from the tissue to the target surface may be influenced by compound-specific physicochemical properties and tissue composition, potentially resulting in differences in transfer efficiency, incomplete transfer, or spatial distortion. A direct comparison between tissue imprinting and cryosection-based MSI was not included in the present study; therefore, the relative advantages and limitations of these approaches cannot be quantitatively established from the current dataset. Finally, the spatial resolution used in the present study allowed interpretation of metabolite distributions at the regional tissue scale but was insufficient for reliable assignment to individual anatomical structures or cellular layers.

Accordingly, the present study should be regarded as a proof-of-concept demonstration of the feasibility of AgNPs-SALDI-MSI for the spatial visualization of low-molecular-weight compounds in *Narcissus* bulb tissue, rather than as a quantitative or fully validated metabolomic study. Nevertheless, the obtained results demonstrate the potential of this approach for rapid spatial metabolomic screening and provide a basis for further methodological development. Future studies involving biological replicates, authentic reference standards, tandem mass spectrometry, quantitative validation, and a systematic comparison with cryosection-based MSI are required to further validate the approach, assess its spatial fidelity and reproducibility, and determine its broader applicability to plant metabolomics and investigations of metabolite localization, plant physiology, and biosynthetic pathways.

## Data Availability

Dataset available on request from the authors.

## Supplementary Materials

The following supporting information can be downloaded at: https://www.mdpi.com/article/10.3390/molecules31172941/s1, Figure S1: Schematic workflow of the AgNPs-SALDI mass spectrometry imaging (MSI) analysis of *Narcissus pseudonarcissus* bulb cross-sections.

## Abbreviations

The following abbreviations are used in this manuscript:

AgNPs	silver nanoparticles
GC–MS	gas chromatography–mass spectrometry
HPLC	high-performance liquid chromatography
LC–MS	liquid chromatography–mass spectrometry
MALDI	matrix-assisted laser desorption/ionization
MS	mass spectrometry
MSI	mass spectrometry imaging
SALDI	surface-assisted laser desorption/ionization
