# Supplementary material for: Silver Nanoparticle-Assisted Laser Desorption/Ionization Mass Spectrometry Imaging of Low-Molecular-Weight Compounds in a Narcissus Bulb
Source: Molecules. 2026 Aug 22;31(17):2941. doi: 10.3390/molecules31172941 (PMC13567441; doi:10.3390/molecules31172941)
Supplement: Supplementary file 1 [file molecules-31-02941-s001.zip › molecules-4489415-supplementary.pdf]

Supplementary Materials

Silver Nanoparticle-Assisted Laser Desorption/Ionization Mass Spectrometry Imaging of Low-Molecular-Weight Compounds in a Narcissus Bulb

Izabela Arendowska [1] and Adrian Arendowski [2,*]

[1] Department of Histology and Embryology, Faculty of Medicine, Medical College, University of Rzeszów, Leszka Czarnego 4, 35-615 Rzeszow, Poland; iarendowska@ur.edu.pl

[2] Faculty of Chemistry, Rzeszów University of Technology, Powstańców Warszawy 6, 35-029 Rzeszow, Poland

* Correspondence: e-mail: a.arendowski@prz.edu.pl, phone: +48 17-865-1541, ORCID: 0000-0002-8496-6800

Figure S1

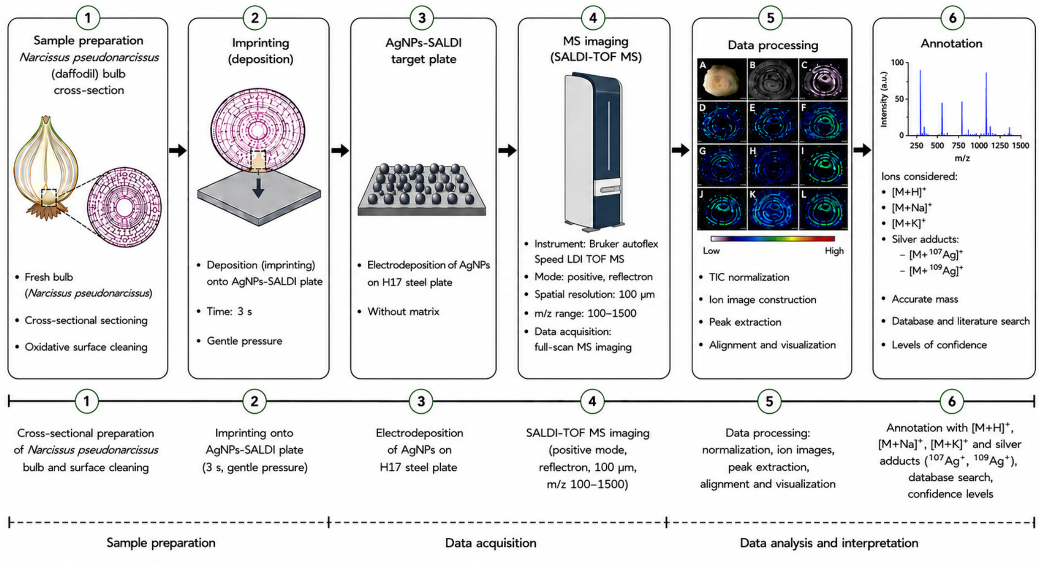

Figure S1. Schematic workflow of the AgNPs-SALDI mass spectrometry imaging (MSI) analysis of *Narcissus pseudonarcissus* bulb cross-sections. The workflow comprises (1) cross-sectional preparation and surface cleaning of the bulb, (2) tissue imprinting onto the AgNPs-SALDI target plate, (3) electrodeposition of AgNPs onto an H17 stainless-steel plate, (4) SALDI-TOF MS imaging using a Bruker autoflex Speed LDI TOF MS in positive reflectron mode, (5) data processing including TIC normalization, ion image reconstruction, peak extraction, alignment and visualization, and (6) metabolite annotation based on accurate mass measurements, characteristic adducts, database and literature searches, and assignment of confidence levels.
